# The Subscription Price of Medical Journals and the Sample Copy Evil

**Published:** 1896-10

**Authors:** 


### THE SUBSCRIPTION PRICE OF MEDICAL JOURNALS AND THE SAMPLE COPY EVIL.

There is a striking variability in the annual subscription price of medical journals. Whether the $1.00 journal is helped by its price is a question. Many subscribers do not pay at all unless pushed. They are, in most cases, not dishonest, but simply negligent. If cash-in-advance were absolutely insisted upon, the $1.00 journal, if of any value, should excel the $2.00 journal in getting subscribers. The pay-in-advance rule, however, is not commonly enforced, and to "credit" many people makes one as cheap to them as the other—they pay neither.

Another difficulty with the subscription business arises from the idea, which is so prevalent, that a publication is able to exist upon its income from advertising. They forget that advertisers have no use for a journal with a "dead-head" subscription list. An honest publisher must have a *bona fide*, paid subscription list. The circulation liar is an important institution in the publishing world, and he often is able to get along without pay subscribers, but he has to hustle to keep from being caught.

At $2.00 per annum a journal of the size and quality of ours is cheap. We may make it $1.00 some day—if the doctors get other journals of equal value for $1.00, and if the subscribers all will pay. It is not what we charge, but what we collect, that has to be considered.

But the greatest drawback to the subscription business is the "sample copy evil." The writer received regularly every week a certain medical publication, properly directed, which he had never ordered and which it took three refusals to stop. That is the kind of circulation which many journals have. No kind of subscription price can meet a donation. An old friend of ours stopped The Journal because he was getting as many sample copies as he could read. A physician gets a sample copy of our Journal about once in seven months. We take care of the regular subscribers first, of the sample copies in proper order.

It seems to us that a thing should have some value, if it ever had, and that even a good thing cannot long be appreciated or received with self-respect if it is given to the recipient. The journal with a definite and just subscription price, should be more valued and more carefully preserved than the journal which is given away, or has a "cut" subscription price.

The rule of The Journal has been to please the subscriber first, holding the old and getting the new by this means, and to act justly and honestly toward advertisers, trusting to the genuineness of our subscription patronage to induce the patronage of advertisers.

M. H. B.

### NOTICE TO SUBSCRIBERS.

Many of you will receive bills for amounts due for subscription. Please read what is printed on these bills and be governed accordingly.

